# Occurrence and Risk Assessment of Aflatoxin M1 in Fermented Dairy Products from the Croatian Market

**DOI:** 10.3390/foods14244354

**Published:** 2025-12-18

**Authors:** Marija Kovač Tomas, Tomislav Rot, Lara Arnautović, Mirjana Lenardić Bedenik, Iva Jurčević Šangut

**Affiliations:** 1 Department of Food Technology, University North, 48000 Koprivnica, Croatia; laarnautovic@unin.hr (L.A.); mlenardicbedenik@unin.hr (M.L.B.); 2 Faculty of Food Technology Osijek, Josip Juraj Strossmayer University of Osijek, Franje Kuhača 18, 31000 Osijek, Croatia; tomislav.rot@inspecto.hr

**Keywords:** aflatoxin M1, fermented dairy products, occurrence, UHPLC-MS/MS, dietary exposure, risk assessment, Croatia

## Abstract

Aflatoxin M1 (AFM1), a hydroxylated metabolite of aflatoxin B1, is a persistent food safety hazard in the dairy production chain. This study investigated AFM1 occurrence in fermented dairy products collected from the Croatian market in spring 2025 and assessed associated dietary exposure risks. A total of 81 samples were analyzed using ultra-high performance liquid chromatography tandem mass spectrometry (UHPLC-MS/MS) following immunoaffinity column clean-up. AFM1 was detected in 48.1% of samples, with a mean concentration of 0.015 µg/kg. Products with thermophilic and probiotic bacterial cultures showed the lowest incidence rates, at 33.3% and 40.0%, respectively. Significantly higher AFM1 occurrence was found in Croatian samples than in imported ones (*p* < 0.05). Exposure assessment, based on estimated daily intake (EDI), hazard index (HI), and margin of exposure (MOE), identified toddlers and children as the most at-risk groups, with EDI ranging from 0.21 to 0.93 ng/kg bw/day, depending on AFM1 concentration. HI exceeded 1 even at mean AFM1 levels, while MOE fell below the safety threshold of 10,000 in worst-case scenarios, indicating potential health concerns. These findings underscore the need for continuous monitoring and targeted risk mitigation strategies for vulnerable populations, and support expanding regulatory limits to include processed dairy products.

## 1. Introduction

Aflatoxins (AFTs) are a group of mycotoxins primarily produced by fungi of *Aspergillus* genus. Known for their potent toxic, mutagenic, teratogenic, and carcinogenic properties, aflatoxins commonly contaminate a variety of foodstuffs and feed, including cereals, peanuts, dried fruits, milk and dairy products. Among them, aflatoxin B1 (AFB1) is considered the most toxic and prevalent. When ingested by lactating animals through contaminated feed, AFB1 is metabolized in the liver into hydroxylated metabolite, aflatoxin M1 (AFM1), which is subsequently excreted in milk [1,2]. According to the International Agency for Research on Cancer, both AFB1 and AFM1 are classified as Group 1 carcinogens [3].

Due to its persistence and potential for further contamination of the food chain, particularly when milk is used as a raw material in the production of other food products, AFM1 contamination represents a significant concern for food safety and public health [1,4]. Accordingly, the European Union (EU) has established maximum acceptable levels for AFM1 by the Commission Regulation (EU) 2023/915, amounting to 0.050 μg/kg in milk, and 0.025 μg/kg in infant formulae and baby foods [5]. However, no specific limits have been set for processed dairy products, despite their widespread consumption in the human diet.

While milk contamination with AFM1 has been extensively investigated over the years [1,6,7,8], fermented dairy products have received considerably less research attention, particularly in regional markets such as Croatia, where available data remain scarce. A recent 20-year data review on the occurrence of AFM1 in the Mediterranean countries highlighted AFM1 as a persistent threat in the dairy sector, emphasizing the need for effective mitigation strategies across all stages of milk production [1]. Moreover, the European Food Safety Authority (EFSA) has identified liquid milk and fermented milk products as major contributors to the dietary AFM1 exposure [9]. According to data from the Croatian Bureau of Statistics, total production of fermented dairy products in 2024 exceeded 193,000 tons, with almost half represented by yoghurts and fermented dairy beverages (https://podaci.dzs.hr/2025/en/97342, accessed on 3 December 2025), highlighting their important role in the Croatian dairy market.

In light of ongoing climate change, aflatoxins have been recognized as one of the foodborne hazards most likely to be affected [10,11]. Long-term shifts in temperature, precipitation patterns, and atmospheric carbon dioxide concentrations are projected to significantly influence the overall occurrence and types of hazards contaminating food [12,13]. In the most probable scenario of a +2 °C increase in environmental temperature over the next century, regions such as Eastern Europe, the Balkan Peninsula, and the Mediterranean are expected to face a rise in aflatoxin contamination [2,14,15,16]. This underscores the increasing importance of monitoring mycotoxin concentrations as an essential part of climate-resilient food safety systems.

Building on these findings, the aim of this study is to conduct a survey of AFM1 contamination in selected fermented dairy products available on the Croatian market and to assess the associated exposure and risk characterization indicators, in order to support evidence-based risk management and inform future food safety regulations related to processed dairy products.

## 2. Materials and Methods

### 2.1. Sample Collection

A total of 81 samples of various fermented dairy products were collected from major Croatian markets during the spring of 2025 (from March to June) for the analysis of AFM1. The products originated from eleven countries: Republic of Croatia (*n* = 49), Germany (*n* = 10), Bosnia and Herzegovina (*n* = 6), Austria (*n* = 5), Slovenia (*n* = 3), Italy (*n* = 3), Belgium (*n* = 1), France (*n* = 1), Poland (*n* = 1), Czech Republic (*n* = 1), and Hungary (*n* = 1). Based on the type of microbial culture used in production, the collected samples were categorized into four groups: mesophilic bacteria (*n* = 10), including sour cream types, sour milk, buttermilk; thermophilic bacteria (*n* = 48), comprising various types of yoghurt, including plain, fruit-flavored, Greek-style, lactose-free, and high-protein varieties; probiotic bacteria (*n* = 10), including probiotic yoghurts, and acidophilus milk; and kefir grains (*n* = 13), encompassing different types of kefir products. Upon arrival at the laboratory, the samples were homogenized, divided into analytical fractions, and stored at −18 °C until analysis.

### 2.2. Chemicals, Equipment, and Analytical Determination

#### 2.2.1. Chemicals, Equipment and Sample Preparation

The analysis of AFM1 was performed using a validated ultra-high performance liquid chromatography tandem mass spectrometry (UHPLC-MS/MS) method, with immunoaffinity column clean-up. Portions of fermented milk samples were weighed into cuvettes (8 ± 0.08 g), and extracted with methanol (22 mL) and ultrapure water (12 mL) for 10 min using a rotator shaker (Bio RS-24, Biosan, Riga, Latvia). Samples were then centrifuged at 4000× *g* for 10 min at 4 °C (LMC-4200R, Biosan, Riga, Latvia), and 30 mL of the supernatant was diluted with 60 mL of ultrapure water. Subsequently, 60 mL of the diluted sample extract was applied onto an immunoaffinity column (AflaStarTM M1 R, Romer Labs, Tulln, Austria) positioned on a vacuum manifold (Chroma Bond, Macherey-Nagel, Düren, Germany), at a speed rate of about 3 mL/min. The column was afterwards washed twice with ultrapure water, and the analyte was finally eluted using 2.5 mL of methanol.

The analytical standard of AFM1 (BiopureTM) was purchased from Romer Labs (Tulln, Austria). Methanol of analytical grade was obtained from Kemika (Zagreb, Croatia), while ultrapure water was generated using Purelab flex system (ELGA LabWater, Wycombe, UK). LC-MS grade formic acid and acetonitrile were supplied by Biovit (Varaždin, Croatia).

#### 2.2.2. Analytical Determination

Instrumental analysis was performed using a Waters UHPLC-MS/MS system consisting of a UHPLC Acquity H-Class (Waters, Milford, MA, USA) equipped with a quaternary pump, coupled to a triple quadrupole mass spectrometer XEVO TQD (Milford, MA, USA) with an electrospray interface. Chromatographic separation was achieved using an Acquity UPLC BEH C18 column, 2.1 × 100 mm, 1.7 µm particle size (Waters, Milford, MA, USA), maintained at 40 °C. A six-minute gradient elution was applied using ultrapure water (mobile phase A) and acetonitrile (mobile phase B), both containing formic acid (0.1%), at a constant flow rate of 0.5 mL/min. The elution program started at 99% mobile phase A, held for 1.0 min, then linearly decreased to 99% mobile phase B at 2.9 min and maintained until 3.7 min, before returning to the initial composition at 3.8 min, held until the end of the run. MS/MS analysis was conducted in multiple reaction monitoring mode, using transition 329.0 > 273.0 *m*/*z* as the quantifier ion and 329.0 > 259.0 *m*/*z* as the qualifier ion. Data acquisition was performed using MassLynx and TargetLynx software (v. 4.1., Waters, Milford, MA, USA).

**Figure 1:**
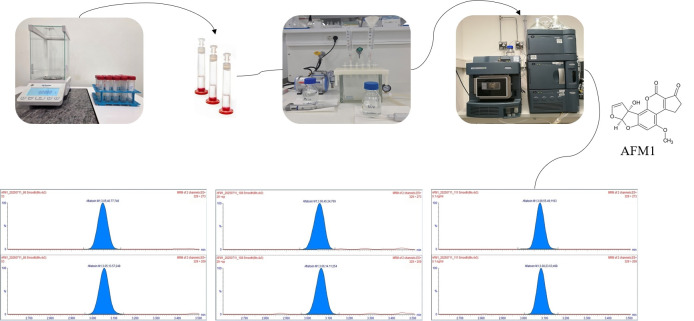
Workflow of AFM1 determination in fermented dairy products.

The method was internally validated and complies with the performance criteria for mycotoxin analysis as specified in relevant EU legislation (Commission Regulation (EC) No 401/2006 [17] and Commission Decision 2002/657/EC [18]). For quantification purposes, external calibration was performed using seven calibrants within a range of 0.02–4.0 ng/mL. Prior to analysis, a blank solvent was injected to equilibrate the system, followed by the calibrants and an additional blank solvent injection before sample analysis to prevent carry-over. For internal quality control within each batch, recovery experiments were performed by spiking the samples with a known concentration of the AFM1 analytical standard. Measured concentrations were corrected for recovery when results fell outside the acceptable range of 90–110%, as specified by Commission Regulation (EC) No 401/2006 [17]. Additionally, at the end of each batch, a calibrant was injected to verify the calibration curve’s suitability and ensure analytical consistency. The limit of detection (LOD) for AFM1, estimated during method validation in accordance with Commission Decision 2002/657/EC [18], was 0.003 µg/kg, while the limit of quantification (LOQ) was 0.010 µg/kg, which were used as threshold criteria for data processing and interpretation. A summarized overview of the whole AFM1 determination procedure is shown in Figure 1.

### 2.3. Dietary Exposure and Risk Assessment

To evaluate the risk associated with the consumption of fermented dairy products, the estimated daily intake (EDI) and hazard index (HI) were calculated. The EDI was determined using the following Equation (1): EDI ng/kg bw/day = AFM1 concentration × daily consumption/body weight (bw). Both mean and maximum AFM1 concentrations were used for EDI calculations. Daily consumption data, defined as the amount of fermented milk product consumed daily per kilogram of body weight, were obtained from the European Food Safety Authority (EFSA) Food Consumption Database [19], which reports chronic consumption of milk and dairy products for various Croatian age groups. For high consumers, data representing the 95th percentile (P95), i.e., the worst-case scenario, were applied. As EFSA data cover the entire milk and dairy products category, a complementary EDI calculation was performed assuming a fixed portion size of 100 g of fermented milk product, with an average body weight of 12.0 kg for toddlers, 26.1 kg for children, 52.6 kg for adolescents, and 70.0 kg for adults and elderly individuals [1]. The HI was calculated using the following Equation (2): HI = EDI/Tolerable Daily Intake (TDI). A TDI value of 0.2 ng/kg bw/day was used, as in similar studies [1,6,7]. Interpretation of HI values was as follows: an HI less than 1 indicates no expected risk or adverse health effects, while an HI greater than 1 suggests potential toxicological concern.

In addition, the margin of exposure (MOE) was calculated to complement the HI approach, in line with the EFSA recommendation for genotoxic and carcinogenic substances such as AFM1 [9]. The MOE was calculated using Equation (3): MOE = BMDL_10_/EDI, where BMDL_10_ is the benchmark dose lower confidence for 10% increase in liver cancer risk. In the absence of a specific BMDL_10_ for AFM1, a potency factor of 0.1 relative to AFB1 was used, yielding in BMDL_10_ of 400 ng/kg bw/day. MOE was calculated for both mean and P95 consumption, obtained using average and maximum AFM1 concentration. MOE values ≥ 10,000 are considered to indicate low public health concern, whereas values < 10,000 suggest potential risk.

### 2.4. Statistical Analysis

AFM1 concentration values were expressed as the mean of three technical replicates ± standard deviation (SD). Statistical analyses were performed using PAST software (version 4.15). A one-way ANOVA followed by Tukey’s post hoc test was applied to determine differences between groups. Statistical significance was considered at *p* < 0.05, and mean values labeled with different letters indicate statistically significant differences. The occurrence of AFM1 in samples from Croatia and those from other countries was compared using a Z-test for proportions. Differences were considered statistically significant at *p* < 0.05, and significantly different results were indicated by different letters.

## 3. Results and Discussion

### 3.1. Occurrence of AFM1 in Fermented Dairy Products

In general, AFM1 levels in dairy products are influenced by multiple factors, including the milk type and quality, particularly the degree of contamination, as well as geographical origin and seasonal variations, processing conditions, and the analytical methods employed for the quantification [1]. Notably, the fermentation process has been shown to contribute to the decrease in the AFM1 concentration due to factors such as low pH, the formation of fermentation-related byproducts, and the activity of lactic acid bacteria, which can reduce AFM1 levels through its binding to the cell wall components or degradation by microbial enzymes [20,21]. For instance, a study investigating AFM1 change during the production and storage of fermented dairy products using various probiotic and non-probiotic cultures demonstrated a significant reduction in AFM1 concentration during fermentation. Among tested cultures, kefir starter was found to be the least effective, whereas probiotic strains and yoghurt cultures proved to be more effective tools for AFM1 reduction [22]. The significance of probiotic bacteria in AFM1 removal from dairy products was further confirmed in a recent study, reporting AFM1 reductions from 75.9 to 96.5% [23]. Consistent with those findings, our results (Table 1) demonstrate the lowest occurrence of AFM1 in fermented dairy products containing thermophilic and probiotic bacterial cultures, with AFM1 detected in 33.3% and 40.0% of the samples, respectively. These occurrence rates were calculated as the percentage of samples with AFM1 levels above the LOD within each product category, serving as a descriptive measure to illustrate the variability among product types available on the market, rather than as a basis for statistical comparison between groups with unequal sample sizes.

To place these results in a broader context, a recent umbrella review of systematic reviews and meta-analyses estimated the overall global prevalence of AFM1 in dairy products at 66.2%, with high heterogeneity among studies, and an average concentration of 0.057 µg/kg across all dairy products. Specifically, available data report an average AFM1 prevalence in yogurt of 58.8% and a mean concentration of 0.047 µg/kg [24]. In comparison, our study found a total AFM1 prevalence in analyzed fermented dairy products to be 48.1%, with a mean concentration of quantified samples of 0.015 µg/kg. These lower values likely reflect more favorable climatic conditions and improved feeding practices, contributing to reduced contamination of raw milk and, consequently, dairy products.

**Figure 2:**
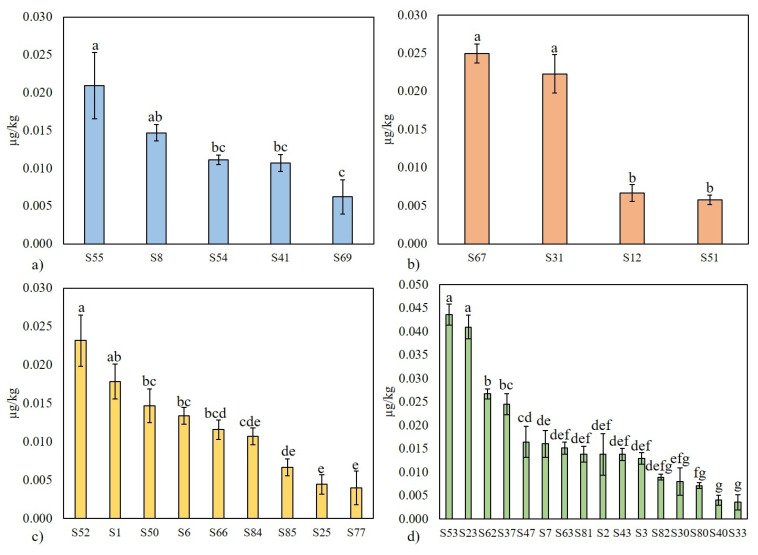
Distribution of AFM1 concentrations across categories of fermented dairy products: (**a**) mesophilic bacteria, (**b**) probiotic bacteria, (**c**) kefir grains, and (**d**) thermophilic bacteria. Values are expressed as mean ± SD (µg/kg). Statistical significance was determined at *p* < 0.05, and values marked with different letters indicate statistically significant differences.

Among individual product categories, distinct patterns in AFM1 contamination were observed. The highest mean AFM1 concentration (0.017 µg/kg) was found in thermophilic culture category, followed by probiotic culture products, mesophilic culture products, and those with kefir grains, as illustrated by the product order: yoghurt > probiotic drinks > sour cream > kefir. In contrast, the frequency of AFM1 detection was the highest in kefir grain category (69.2%), followed by mesophilic culture products, probiotic products, and thermophilic culture category, as represented by the product order: kefir > sour cream > probiotic drinks > yoghurt. Distribution of AFM1 concentrations across categories of fermented dairy products is shown in Figure 2.

In contrast, milk contamination has been widely investigated both globally and in Croatia, with a well-established association to AFB1 contamination of animal feed, and higher levels typically observed during the autumn and winter seasons [1,6,7,8]. A recent national study on raw milk collected between 2022 and 2024 reported that 15.1% of samples exceeded the EU threshold, with the highest incidence recorded in autumn 2024 [6]. In addition, a mini-survey on ultra-high temperature (UHT) milk contamination originating from 2022 detected AFM1 in 93.8% of samples, with an average concentration of 0.019 µg/kg [25]. A previous study analyzing 5817 raw milk samples collected between 2016 and 2022 found 3.5% of samples were contaminated, with the highest incidence of positives in winter 2019/2020 [7]. Earlier investigations, during the milk crisis period of 2013–2014, showed substantially higher contamination. For instance, the analysis of over 4400 milk samples collected during the first half of 2013 revealed AFM1 levels exceeded the EU threshold in 27.8% of raw and 9.6% of UHT milk samples, reflecting optimal weather conditions for AFB1 synthesis in raw feed material and subsequent carry-over into milk [8]. When looking globally, AFM1 prevalence in raw and processed milk has been estimated to range from 64.8% to 88.7% [24]. Meanwhile, a 40.0% contamination rate was reported in Mediterranean countries, regardless of milk type, further highlighting the importance of continuous AFB1 monitoring, as well as strict control of processing and storage conditions to minimize risk for consumers [1].

Furthermore, given that 60.5% of the analyzed samples originated from Croatia, a comparative analysis of AFM1 occurrence based on product origin (Croatia or non-Croatia) was conducted to assess significant differences in contamination levels. In recent decades, Southeastern Europe, including Croatia, has experienced climate change effects characterized by rising average air temperature, more frequent summer droughts, and occasional episodes of extreme precipitations [4,26]. These shifts towards warmer and drier conditions are already impacting agricultural production by increasing the plant’s susceptibility to infection and consequent mycotoxin contamination, as well as altering the geographical distribution of toxigenic fungi [14,27]. Environmental temperature, together with water activity, is considered the main contributor to fungal growth and mycotoxin biosynthesis. Generally, *Aspergillus* species thrive under conditions of low water activity and elevated temperatures, with optimal aflatoxin production occurring at approximately 33 °C and a water activity of 0.99 [12,28,29]. Although traditionally dominating in warmer parts of the world, primarily tropical and subtropical climates, aflatoxin-producing fungi are now increasingly expected to contaminate crops in central [15,16,29].

Comparative analysis (Table 2) of Croatian and imported products revealed a significantly higher proportion of AFM1-positive samples in products of Croatian origin (*p* < 0.05), with occurrence rates of 55.1% and 21.9%, respectively. These differences may be primarily attributed to the increasingly warm and dry climate in Croatia, which creates favorable conditions for the growth of *Aspergillus* species in feed crops such as maize, more so than in cooler, wetter regions. According to the Croatian Meteorological and Hydrological Service [30], July and August 2024, critical months for maize infection, were the hottest on record, while large parts of the country simultaneously experienced normal to below-normal precipitation levels, with several regions affected by dry to extremely dry conditions. These climatic extremes likely promoted fungal proliferation and aflatoxin biosynthesis in crops, reflected by elevated AFM1 levels in fermented dairy products. When analyzed by individual country of origin, AFM1 was detected in some samples from neighboring countries, but was nearly absent in samples originating from countries located north of Croatia. These contamination patterns support the premise that climate-driven factors enhance aflatoxin biosynthesis in Croatian agricultural systems, facilitating its transfer from contaminated feed to milk and dairy products.

### 3.2. Dietary Exposure and Risk Assessment for Different Age Groups

Based on the established link between AFB1 contamination in feed and subsequent AFM1 presence in dairy products, as well as the toxicological relevance of AFM1, this study assessed the dietary exposure and potential health risk to Croatian consumers through the consumption of selected fermented dairy products. Estimated dietary exposures, expressed as EDI, and risk characterization, expressed as HI, for different age groups are summarized in Table 3.

EDI was calculated using both the mean AFM1 concentrations of 0.015 µg/kg and the maximum found concentration of 0.044 µg/kg. The highest exposure estimates were observed in toddlers and children, with EDI values ranging from 0.21 to 0.93 ng/kg bw/day, depending on the applied concentration. For high dairy consumers, P95 consumption data were used, resulting in substantially elevated EDI values across all age groups. As expected, toddlers exhibited the highest exposures, particularly when the maximum AFM1 concentration was considered, with EDI values reaching 0.74 and 2.17 ng/kg bw/day, respectively.

The risk of exposure through fermented milk consumption was further assessed using HI values, calculated as the ratio of EDI and a safety threshold of 0.2 ng/kg bw/day [1,6]. Using the mean AFM1 concentration, toddlers and children exhibited the highest dietary risk, with HI exceeding 1 (1.58 and 1.03, respectively), primarily due to their lower body weight and higher relative consumption. HI values increased substantially when the maximum AFM1 concentration was used, reaching up to 4.64. The worst-case scenario, combining the P95 consumption with the maximum AFM1 concentration, resulted in HI values ranging from 1.26 to 10.83, in the following order: toddlers > children > adolescents > very elderly > adults > elderly, indicating a significant health concern across all age categories, with young children being at the highest risk. Results from a comparative analysis using a fixed portion size of 100 g for EDI and HI calculations showed a similar age-dependent risk pattern, supporting the observed trend of elevated intake risk among younger populations.

These exposure and risk patterns align with previous studies. Since the EDI and HI indicators are highly dependent on AFM1 concentrations in dairy products, which are largely influenced by climatic factors, regions characterized by warm and dry conditions, such as the Southeastern Mediterranean countries, are considered to be at the highest risk [1]. An assessment of AFM1 intake from milk and yoghurt among student populations in Serbia and Greece revealed average exposures between 1.24 and 2.67 ng/kg bw/day for Serbia, and 0.35 to 0.450 ng/kg bw/day for Greece, with corresponding HI values suggesting elevated risk, particularly in Serbia [31]. In Croatia, although this study focused on fermented dairy products, recent investigations on raw milk samples have shown similar AFM1 contamination levels and associated risk indicator estimations. Toddlers and children exhibited the highest mean EDI values (0.61–0.67 ng/kg bw/day and 0.41–0.43 ng/kg bw/day, respectively), indicating elevated risk of exposure [6].

Furthermore, several African and Asian countries have also reported high AFM1 prevalence and elevated HI values. For example, a recent study from Iran found AFM1 in 85.6% of raw milk and dairy products, with a mean concentration of 0.251 µg/kg and 68.9% of samples exceeding the EU regulatory limit. Among tested products, yoghurt was identified as the greatest contributor to AFM1 exposure due to its high consumption rates, resulting in HI values above 1 across the population [32]. In India, mean AFM1 concentrations ranged from 0.100 to 0.321 µg/kg, depending on the product type, with corresponding EDI values of 0.02–2.30 ng/kg bw/day, and highest risk observed in children [33]. Similarly, a study conducted on raw milk and cheese in Ethiopia reported mean HI values between 0.27 and 7.05, depending on the region, indicating potential health concerns, especially among high milk consumers [34]. These findings collectively reflect the variability of AFM1 exposure and associated health risk across different regions and population groups.

**Figure 3:**
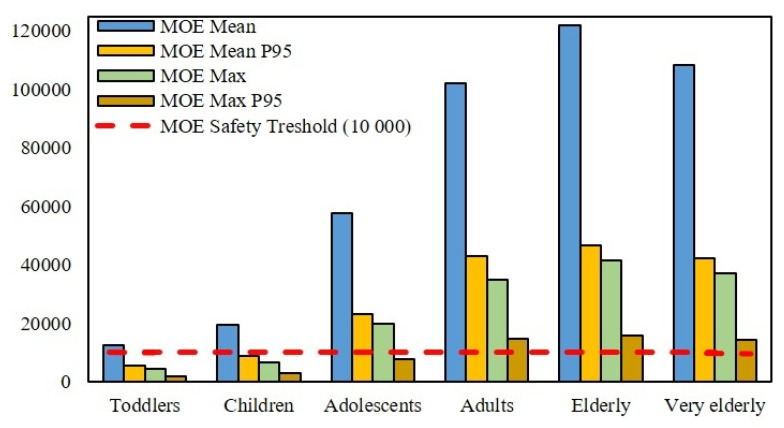
MOE risk assessment for AFM1 in fermented dairy products by age group in Croatia.

The MOE-based risk characterization further complements the overall findings of our study, providing an additional perspective on the potential health risks associated with AFM1 exposure. Figure 3 shows the calculated MOE values across age categories, most of which exceed the critical threshold of 10,000. However, lower values observed in the youngest age groups point to a potential health concern.

The lowest MOE values are found in toddlers and children under the worst-case scenario (P95 consumption combined with maximum AFM1 concentration), reaching 1846 and 3032, respectively. These results are consistent with the HI-based risk assessment (Table 3) and align with previous risk characterization studies on milk and dairy studies [6,9,35,36,37]. Although high exposure to AFM1 from milk and dairy products is generally limited to a short period of life [9], considering climatic variability affecting AFB1 contamination of feed and subsequent AFM1 synthesis, continuous monitoring and periodic updates of exposure assessments remain essential.

## 4. Conclusions

To the best of our knowledge, this study provides the first comprehensive and recent data on AFM1 contamination in fermented dairy products (including yoghurt, kefir, sour cream, and similar) from the Croatian market, highlighting the influence of microbial cultures and geographic origin on toxin prevalence. AFM1 was detected in nearly half of all samples, with most contamination levels remaining below the EU maximum limit for milk (0.050 µg/kg). However, products of Croatian origin exhibited significantly higher occurrence rates than imported ones, while AFM1 occurrence variability was observed across the different categories of fermented dairy products. Dietary exposure assessment identified toddlers and children as the most vulnerable groups, with HI values exceeding 1 even at mean AFM1 levels and MOE values below the safety threshold of 10,000 in worst-case scenarios, indicating a potential health concern. These findings underscore the importance of continuous monitoring and targeted mitigation strategies to further minimize risks to susceptible populations. Moreover, the results reveal limitations in current EU legislation, which regulates AFM1 only in milk, emphasizing the need to expand surveillance and implement stricter control measures across a broader range of dairy products, particularly given the increasing mycotoxin risks associated with ongoing climate change.

## Figures and Tables

**Table 1:** Mean concentrations (± SD) and occurrence (%) of AFM1 in different categories of fermented dairy products according to microbial culture.

Microbial Culture	*n* *	Mean ± SD µg/kg	Occurrence %
Mesophilic bacteria	10	0.013 ± 0.005	50.0
Thermophilic bacteria	48	0.017 ± 0.012	33.3
Probiotic bacteria	10	0.015 ± 0.010	40.0
Kefir grains	13	0.012 ± 0.006	69.2

* *n*—number of samples per product category.

**Table 2:** AFM1 occurrence (%) in fermented dairy samples according to product origin.

Product Origin *	*n*	Mean ± SD µg/kg	Median µg/kg	Occurrence %
Croatia	49	0.015 ± 0.010	0.014	55.1 ^a^
Non-Croatia	32	0.012 ± 0.007	0.012	21.9 ^b^

* Comparison was made between samples from Croatia and those from other countries. The number of samples per product origin category (*n*) is shown, and both the mean (±SD) and median values are expressed in µg/kg. Different letters indicate statistically significant differences (*p* < 0.05).

**Table 3:** EDI and risk assessment of AFM1 exposure through fermented dairy consumption for different age groups in the Croatian population.

Age Category	Data Type	Consumption g/kg bw/day	0.015 ng/kg AFM1 ^a^		0.044 ng/kg AFM1 ^b^	
			EDI	HI	EDI	HI
Toddlers 1–3	EFSA Mean ^c^	21.11	0.32	1.58	0.93	4.64
	EFSA P95	49.24	0.74	3.69	2.17	10.83
	Calculated ^d^	8.33	0.13	0.63	0.37	1.83
Children 3–10	EFSA Mean	13.77	0.21	1.03	0.61	3.03
	EFSA P95	29.98	0.45	2.25	1.32	6.60
	Calculated	3.83	0.03	0.29	0.17	0.84
Adolescents 10–18	EFSA Mean	4.62	0.07	0.35	0.20	1.02
	EFSA P95	11.56	0.17	0.87	0.51	2.54
	Calculated	1.90	0.02	0.14	0.08	0.42
Adults 18–65	EFSA Mean	2.61	0.04	0.20	0.11	0.57
	EFSA P95	6.22	0.09	0.47	0.27	1.37
	Calculated	1.43	0.02	0.11	0.06	0.31
Elderly 65–75	EFSA Mean	2.19	0.03	0.16	0.10	0.48
	EFSA P95	5.72	0.09	0.43	0.25	1.26
	Calculated	1.43	0.02	0.11	0.06	0.31
Very elderly >75	EFSA Mean	2.46	0.04	0.18	0.11	0.54
	EFSA P95	6.29	0.09	0.47	0.28	1.38
	Calculated	1.43	0.02	0.11	0.06	0.31

^a^ Mean AFM1 concentration; ^b^ Maximum AFM1 concentration; ^c^ Data from the EFSA Food Consumption Database; ^d^ Data calculated using a fixed portion size of 100 g (see Section 2.3).

## Data Availability

The original contributions presented in the study are included in the article; further inquiries can be directed to the corresponding authors.
